# How college experiences impact student learning outcomes: Insights from Chinese undergraduate students

**DOI:** 10.3389/fpsyg.2022.1021591

**Published:** 2022-11-08

**Authors:** Hua Bai, Hongwei Yu, Rogerland Bantsimba N., Li Luo

**Affiliations:** ^1^School of Education, Minzu University of China, Beijing, China; ^2^Center for Community College Student Engagement, University of Texas Austin, Austin, TX, United States; ^3^College of Preschool Education, Capital Normal University, Beijing, China

**Keywords:** undergraduate education, student learning outcomes, social and academic activities involvement, structural equation modeling, China

## Abstract

In recent years, student development and learning outcomes have become a central focus of quality higher education research. Identifying factors associated with student learning outcomes is the key to continuous quality improvement. The purpose of this study was to explore the impact of college experiences on student learning outcomes. A sample of 17,609 undergraduates from 23 Chinese colleges/universities were selected for the study and asked to complete the Chinese version of the College Student Experiences Questionnaire. Structural equation modeling analyses suggested academic and social activity involvement was an important factor influencing student learning outcomes. Campus facilities and the college environment also predicted student learning outcomes. Family background, on the other hand, exerted minimal influence on student learning outcomes. Students’ involvement in academic and social activities served as strong mediators between campus facilities/environment and student learning outcomes. The results highlighted the significance of providing high-quality college experiences. They also informed policymakers and practitioners of possible pathways to support student learning outcomes by enhancing students’ involvement in social and academic activities.

## Introduction

From a global perspective, higher education is an important pursuit involving multiple stakeholders, such as government agencies, social and business organizations, and parents. Quality education is a lifeline for higher education; providing and pursuing quality higher education is a major goal for universities and students, respectively. The outcomes for students enrolled in higher education institutions (HEIs) have long been of interest to educational scholars and policymakers. In China, the enrollment expansion policy of higher education has been in place since 1999, and today, higher education has entered the era of massification. The gross enrollment rate in HEIs has increased from 9.76% in 1998 to 54.4% in 2020; the number of HEIs has increased from 1,022 in 1998 to 2,738 in 2020; and enrollments have increased from 1,156,100 in 1998 to 14,536,000 in 2020 (Ministry of Education of the People’s Republic of China, 2021). However, educational quality has become an issue. Rapid expansion in the number of Chinese college students has not been accompanied by a proportionate increase in resources. The average size of regular HEIs has gradually increased, which has led to insufficient campus facilities and a shortage of teachers. How to transform from scale expansion to quality improvement has become a top priority.

When discussing the quality of education, it is very important to understand the concept of quality. The original concept of quality is imprinted in the fields of economy and management, and its essence refers to excellence, which can be used to judge the degree of excellence of the object to be evaluated (Borden, 2011). With the rise of value philosophy, value and value judgment have become the guiding ideology governing the concept of quality; as a result, quality is defined from the customer’s perspective. Quality refers to the fitness for intended use or conformance to requirements, which implies the degree of satisfaction and compliance with the object (Shi and Wang, 2010). With the entry of multiple stakeholders into the university field (including the government, enterprises, alumni, donors, social media, etc.), the current multi-stakeholder pattern has gradually formed. Therefore, the essence of quality becomes the question of value selection, and the quality demands put forward by these different stakeholders become an important dimension to measure the quality of education. As the core function of higher education, the quality of talent training should be considered in the macro higher education system, and students are at the center of the whole system. Due to the unique attributes of the student subject, the quality of higher education should not only meet the needs of the student subject but also have the student as an intermediary to connect multiple stakeholders and meet the needs of other subjects. Therefore, the training specifications of students have become an important standard for measuring the quality. How to assess the development of students, including the process and outcome, is the key to understand the concept of educational quality.

In the effort to improve the quality of higher education, the Chinese government attaches great importance to building world-class universities that are globally competitive. After the central government successively launched the “211 Project” and the “985 Project,” it put forward the policy of “World-class University and First-class Discipline Construction” in 2017 and promoted the construction of key universities and disciplines in higher education. It also conducted the “National Undergraduate Teaching and Learning Quality Assessment,” concurrently, focusing on the assessment of enrollment, student guidance, and learning and employment quality based on student development. From the perspective of the development history of universities, talent training is the essential function of universities, and undergraduate education serves as a foundation for universities. World-class universities have placed undergraduate education in an important strategic position for development, and have taken cultivating first-class undergraduates as a critical goal. Undergraduate education is the main body of higher education in mainland China and occupies the central position in the structure of higher education. For a long time, undergraduates have accounted for the majority of the total number of students in Chinese HEIs, and the focus of educational policies is on the undergraduate education. In general, the primary goal of undergraduate education is to implement general education for students, with a focus on improving students’ overall quality and all-round development. In order to meet the needs of the future society, its aim is to enhance students’ learning ability, develop their analytical skills, increase their ability to process new information, and come up with independent conclusions (Casey, 2004). Studies on the development of undergraduate students have become the focus of higher education research, and student learning outcomes are considered a crucial component. Researchers have gradually paid attention to investigate the quality of talent training from the perspective of educational outcomes. During quality evaluation, as an important dimension, learning outcomes serve as the basis for measuring students’ educational outcomes.

Thus, in this study, we focused on how undergraduate education impacts student learning outcomes and asked the following questions: what kind of factors influence student learning outcomes in college? How does the college learning experience influence and promote the acquisition of learning outcomes and what are the underlying mechanisms? The answers will have important theoretical and practical significance.

### Student learning outcomes and their influencing factors

While higher education achieves the growth of quantity and scale, it also brings hidden concerns about quality. The public attention and research on quality have begun to increase, and the theme of quality has become the core issue of higher education. In recent years, scholars have carried out research on the quality of higher education, including investigating both “whose quality view” and “what kind of quality view.” Views on the quality have shifted from epistemology to axiology, and the quality landscape dominated by multiple stakeholders includes as many quality concepts as possible (Shi and Wang, 2010). Tracing the concept of quality from the policy context, the inquiry of quality has moved from behind the scenes to the front, from the prescriptive quality concept to the demand-oriented quality concept, and then to the emphasis on the developmental quality concept, and the essence of quality has shifted from exogenous demand to endogenous development (Hu, 2006). The diversified interest demand pattern formed in the field of higher education makes the view on higher education quality need to be built on the basis of systematic thinking, and students, as the most important stakeholders, could connect different stakeholders (Borden, 2011). In the evaluation of educational quality, emphases have placed on exploring the evaluation system with student learning outcomes as an important dimension, which focuses on students’ learning experience and learning involvement, and investigates the learning environments universities provide for students, as well as students’ adaptation and integration status (Hu and Kuh, 2003; Pascarella et al., 2011; Lv, 2020).

Student learning outcomes are the most important dimension in higher education quality assessment and are an important basis for measuring overall educational outcomes. Similar concepts, such as learning outcomes, student gains, college outcomes, and individual development, have been explored in previous studies (Hu and Kuh, 2003; Borden, 2011; Brown et al., 2013). Currently, no consensus has been reached on the definition of student learning outcomes. It is essentially the outcomes obtained after participating in certain forms of study, which may or may not be intentional (Davis and Murrell, 1993). The current college education system places great emphasis on outcome-based or standards-based education. By defining learning outcomes, they can be woven into professional and project learning activities. Learning outcomes include both knowledge and transferrable skills, and specify both what students ought to learn and what they can do with what they have learned (Casey, 2004). Thus, it can be inferred that student learning outcomes mean students can demonstrate their knowledge, skills, and values upon completing a series of courses or training programs (Hu and Kuh, 2003).

A large body of empirical studies has uncovered the effects of multifaceted factors on student learning outcomes and their sub-dimensions (Topping, 1996; Terenzini et al., 1999; Shi et al., 2020; Lucero et al., 2021). These multifaceted factors include background characteristics, institutional and external college environmental factors, and more importantly, students’ involvement in different types of learning activities (e.g., Pascarella and Terenzini, 2005; Kuh, 2009; Mayer et al., 2019). These related studies specifically include, students’ pre-college characteristics such as gender, family income, parental education background, and parental involvement are closely related to their learning outcomes but these factors only have conditional effects (Pike and Kuh, 2005; Hill and Chin, 2018; Clever and Miller, 2019). College-level factors not only affect student learning outcomes but also student engagement in academic studies. The hardware level of institutional structures, institutional density, differentiation of the curriculum, and clear and organized classroom instruction have become important factors that impact student learning outcomes and degree completion (Osegura and Rhee, 2009; Xerri et al., 2018; Monson, 2019). The quality of campus relationships, the interpersonal environment, and support structures for students’ success have positive correlations with student development outcomes (Mahan, 2010; Pascarella et al., 2011; Pak, 2020).

In addition, there is extensive research on the relationship between involvement in various types of social and academic activities and student development. These activities and input factors have positive impacts on student learning outcomes. Students often use in-class discussions, group activities, out-of-class learning, writing assignments, and online learning and discussion during the learning process. These activities exert positive impacts on students’ critical thinking skills and knowledge integration and application (Mckinney et al., 2004; Howard and Zoeller, 2007). Students’ activities such as involvement in any forms of collaboration, effective interaction with teachers, active participation in extracurricular activities, formation of student learning communities, and active discussion with team members have a positive influence on student learning outcomes (Butler and Dawkins, 2008; Keen and Hall, 2009; Laird and Cruce, 2009). Furthermore, types of student engagement and levels of active learning have positive impacts on the development of students’ generic competency (Choi and Rhee, 2009; Yu et al., 2011).

### College experiences and college impact models

Several college impact models assume that students who put more time and energy into learning-related activities are more likely to develop academically. The more effort students devote to these activities, the more benefits they are likely to obtain. For instance, Feldman and Newcomb (1969) reviewed decades of early college impact studies, including both empirical and theoretical studies, and found that college exerts specific effects on students’ determination to graduate. Chickering and Gamson (1987) proposed seven principles of good practice for undergraduate education, such as academic and social communication with teachers, participation in student clubs and organizations, participation in research projects, and so on. Pace (1979) proposed a student input model and hypothesized that the time and effort students put into their college experiences, along with their use of campus facilities and opportunities, are related to student learning outcomes. Astin (1993) proposed the Input-Environment-Output model, which hypothesizes that student input, including a series of experiences students encounter after entering college, influences student development outcomes in the college environment. Tinto (1975) developed a model for college dropout syndrome and pointed out that students are most likely to drop out in the first year of college when their academic and/or social integration is still uneven. Pascarella (1985) proposed a comprehensive college impact model in which the external environmental variables, organizational characteristics, and student background characteristics, impact individual student learning and cognitive development, mediated by students’ social interaction, personal efforts, and the college environment. Weidman (1989) proposed an undergraduate socialization model, which hypothesized that college students’ development is influenced by family socialization, pre-college enrollment characteristics, and college experiences. The learning productivity model proposed by Kuh and Hu (2001) focuses on the influence of university from the perspective of student input, especially self-input and school input. According to these models, these college experiences are effective and can promote student learning and development in various types of institutions of higher education. Researchers have examined student development by using empirical data collected from questionnaires [e.g., College Student Experiences Questionnaire (CSEQ) and the National Survey of Student Engagement] and identified several important factors affecting student learning and personal development. These factors include an early emphasis on extracurricular learning experiences, involvement in extracurricular activities, faculty-student interaction, and student collaboration (Kuh, 1995; Kuh et al., 1997). Researchers have also studied the effects of demographic characteristics, college environment, and academic support on student learning outcomes and found them to be directly related to the quality and quantity of the time and effort students invest in educational activities (Kuh and Hu, 2001; Hu and Kuh, 2003). In their classic book *How College Affects Students*, Pascarella and Terenzini (2005) reviewed existing college impact studies and concluded that the extent to which the college affects students is, in large part, determined by students’ input and efforts.

### Student learning outcomes in Chinese colleges and universities

Since the expansion of higher education enrollment in the late 1990s, the number of students enrolled in HEIs, and the scale of enrollments in China have expanded continuously. As more challenges have emerged in undergraduate education, the Chinese government has gradually strengthened its reform and support for undergraduate education and has successfully formulated relevant educational policies. In 2018, the Ministry of Education formulated the policy “Opinions on Accelerating the Construction of High-level Undergraduate Education and Comprehensively Improving the Ability to Cultivate Talents” that also included an “Undergraduate Education-Oriented Development Concept” with the following goals—to comprehensively revitalize undergraduate education, focus on student development, strengthen the management of the learning process, and highlight the level and quality of talent training based on an evaluation of their performance.

In recent years, the introduction of questionnaires to gather data on learning engagement and student development has greatly influenced studies on undergraduate development in China. Among the most widely used survey tools are the Chinese version of CSEQ, the China College Student Survey, and Student Experience in Research University. The survey respondents included undergraduates at both research universities and baccalaureate colleges. In these studies, student background characteristics, learning engagement, and involvement in various types of activities have similar positive effects on learning outcomes and their sub-dimensions (Lu and Li, 2020; Lv, 2020; Qin and Lv, 2022). Specifically, factors at the institutional level include institutional structures, quality of teaching, the external classroom learning environment, campus atmosphere, campus culture, and college environment (Bai and Liu, 2017; Zhang and Tang, 2021; Ma and Feng, 2022; Zhuang and Liu, 2022). The factors at the student level include personal characteristics, faculty-student interaction, peer interactions among students, library experiences, course learning, academic participation, and research input (Guo and Han, 2018; Lv, 2021; Shi, 2022). It is through the interaction of student input and institutional investment that students can achieve personal growth and development at college.

### The current study

In college, student development can be conceptualized as a “black box” in that it is a long and complicated process and is impacted by multiple and multifaceted factors. To date, researchers have made substantial efforts to identify these factors based on existing models. In most of these studies, researchers attempted to verify and revise the models by using empirical survey data, and employed a variety of quantitative data analysis methods to explore the influence of various factors on student learning outcomes (e.g., Xerri et al., 2018; Monson, 2019; Pak, 2020; Qin and Lv, 2022; Zhuang and Liu, 2022). Student and institutional level factors most likely to impact students’ development outcomes were identified, including student characteristics, learning engagement, participation in curricular and extracurricular activities, the external environment of the college, and campus facilities. Yet, existing theoretical models have not been sufficiently tested and the inter-relationships among influencing factors are under-explored.

Previous studies did not focus on identifying the possible mediating effects of the factors relevant to student learning outcomes. As much of the research on college impact models and factors associated with student development are from the USA, their findings may not be applicable in the Chinese context given the significant differences between the campus culture in China and the USA. Moreover, undergraduate students in China may have had dramatically different college experiences than undergraduate students in the USA. Therefore, the purpose of this study is to explore the role that Chinese universities/colleges play in undergraduate students’ learning and development. To that end, we specifically addressed the following questions: (1) To what extent are undergraduate students’ background characteristics, involvement in college activities, and external campus conditions associated with their learning outcomes? and (2) Does students’ involvement in college activities mediate the relationship between students’ background characteristics, the external campus conditions, and students learning outcomes?

## Materials and methods

### Conceptual framework

The conceptual framework guiding this study is adapted from college impact models from previous studies. Pace (1985) underscored the direct impact of student background and college environmental factors and the indirect impact of student effort on learning outcomes. Astin (1993) focused on the direct impact of student background, personal characteristics, and pre-college learning experience on student learning outcomes. Tinto (1975) highlighted the direct impact of student background and pre-college education and the indirect impact of academic and social activities involvement on student learning outcomes. Pascarella (1985) stressed the direct impact of student background and campus facilities, and the indirect impact of individual student effort and interpersonal interactions on student learning outcomes.

**Figure 1:**
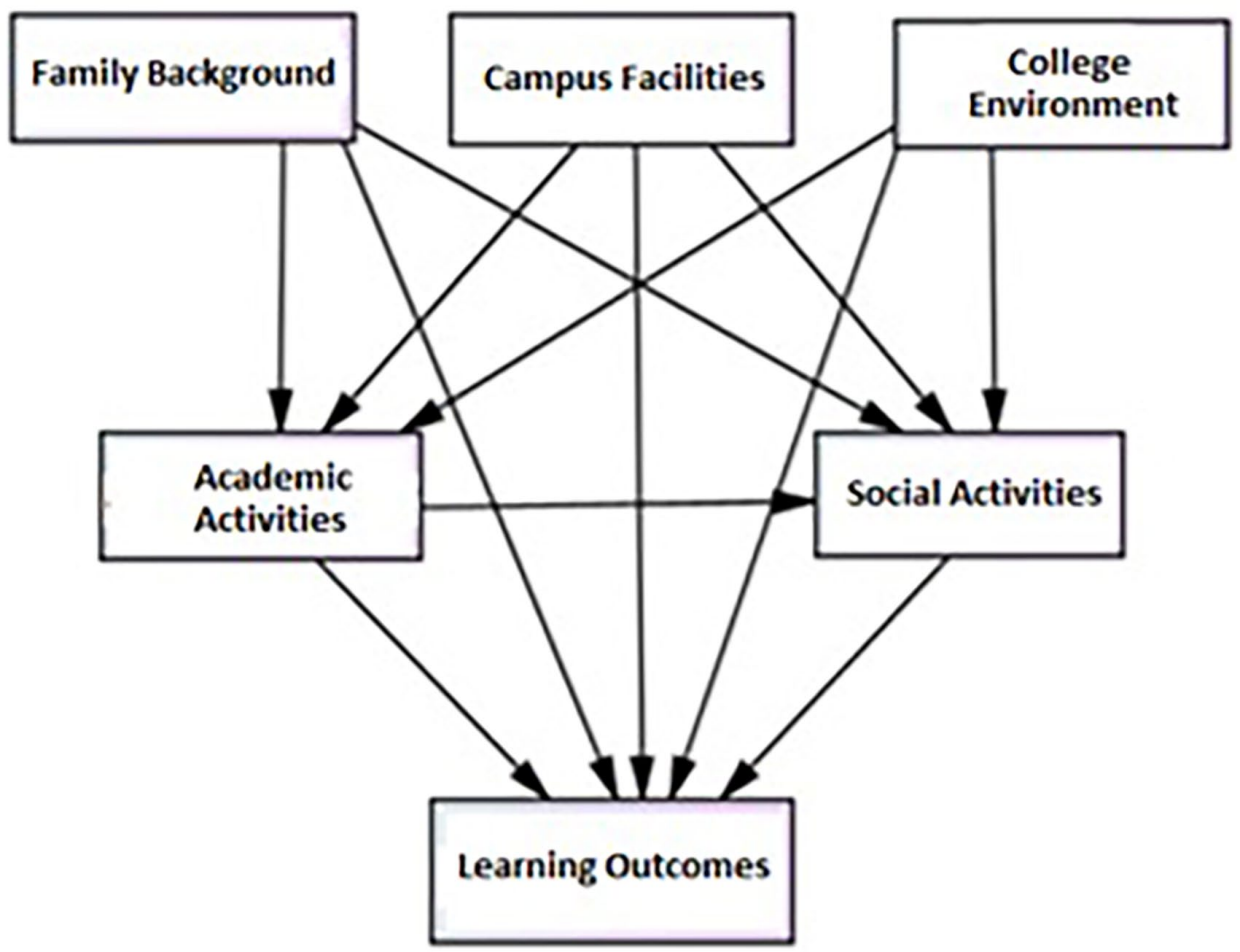
The proposed college impact model.

Informed by the findings of previous studies, our conceptual framework (see Figure 1) hypothesized that student background characteristics, their involvement in various activities, the external college environment, and campus facilities would impact student learning outcomes. This model can be constructed according to the logical structure of input (exogenous), process (endogenous), and output (results) as per Astin’s I-E-O model (1993). The variables for INPUT include family background, college external environment, and campus facilities. The variable for PROCESS includes personal effort reflected in students’ involvement in various types of activities. The participative activities may be classified as academic activities and social activities. The variable for OUTPUT is the learning outcomes that students achieve through a college education.

When constructing impact paths, students’ family background, college environment, campus facilities, involvement in academic activities, and social activities have a direct impact on student learning outcomes. Meanwhile, family background, the college environment, and campus facilities have an indirect impact on student learning outcomes through involvement in academic and social activities. Concerning the relationship between involvement in academic activities and social activities, studies show that students’ academic integration has a greater impact on students’ development when combined with social integration, in comparison to their development through academic integration alone (Tinto, 1975; Davis and Murrell, 1993).

### Participants

China ranks third in the world by total land area, and there exist growing economic disparities across the eastern, central, and western regions. This has also brought gaps in the development of universities across different regions. The number of universities located in the eastern region is much larger than those in the central or western regions. Furthermore, the Chinese central government has invested more in some universities, such as universities included in the “211 Project” or “985 Project.” Therefore, we considered both the region and type of universities in selecting the participants. Firstly, we selected 16 universities from the eastern region, four universities from the central regions, and three universities from the western regions. Secondly, we selected three “985 Project” universities, three “211 Project” universities, and 17 universities that are not included in either “985 Project” or “211 Project.” A total of 23 Chinese universities and colleges from Beijing, Shandong, Inner Mongolia, and Xinjiang provinces were selected for this study. Undergraduates enrolled in these universities and colleges were randomly selected to respond to the survey. The undergraduate sampling accounts for different types of universities/colleges (e.g., research-oriented, teaching-oriented), geographical location of universities/colleges (eastern, central and western), grade categories (freshmen to senior), and ethnic categories (Han or minority group students), which can better represent Chinese undergraduates. A total of 18,473 students were invited to participate in this study and 17,609 returned the survey, indicating a 95.32% response rate. The distribution of undergraduates’ responses by location of universities/colleges are as follows: n = 6,850 (38.90%) from Beijing; n = 6,502 (36.92%) from Shandong; n = 2,295 (13.03%) from Inner Mongolia; and n = 1,962 (11.15%) from Xinjiang.

The samples were also divided by types of universities/colleges as follows: *n* = 6,459 (36.68%) from the “985 Project”^1^ (i.e., research-oriented) universities; *n* = 3,896 (22.13%) from “211 Project” (i.e., research-oriented) universities; and *n* = 7,254 (41.19%) from teaching-oriented colleges. The distribution of responses by grade was as follows: *n* = 4,598 (26.11%) freshmen; *n* = 5,633 (31.99%) sophomore; *n* = 4,129 (23.45%) junior; and *n* = 3, 249 (18.45%) senior. There were 7, 252 (41.18%) male students and 10,357 (58.82%) female students in the sample. Among these students, 13,931 (79.11%) were students from the Han group and 3,678 (20.89%) were from minority groups. Concerning their majors, 6,713 (38.12%) were from the disciplines of the humanities and social sciences (education, literature, history, philosophy, law, and sociology), 2,477 (14.07%) were from the disciplines of economics and management (economics, management, and business), 7,408 (42.07%) were from the disciplines of the natural sciences (science, engineering, agronomy, and medical science), and 1,011 (5.74%) were from the discipline of the arts and sports (music, art, sports, and fine arts).

### Measures and variables

The Chinese version of the CSEQ (Pace, 1979) was used to collect empirical data from students enrolled at Chinese HEIs. The CSEQ was developed by Robert Pace at the University of California Los Angeles in the 1970s. It was revised three times before the research program was moved to Indiana University Bloomington under the leadership of George Kuh in 1994. Pace and Kuh coauthored the fourth and current edition of the CSEQ. The questionnaire has been in existence for 50 years and revised four times in the United States; each time it obtained adequate reliability and validity scores. In 2001, the CSEQ was introduced in China by professor Zuoyu Zhou at Beijing Normal University (BNU). The CSEQ was translated into simplified Chinese and minor revisions were made on the Chinese version of the CSEQ (Zhou and Zhou, 2012). According to the actual situation of Chinese universities, revisions were made on the content of activities that students participate in. For example, in the dimension of library experiences, one item related to searching for scholarly literature written in a foreign language (e.g., English) was added; in the dimension of computer and information technology, one item related to attending online courses offered by universities outside China was added. The Chinese version of the CSEQ has adequate psychometric properties (Zhou and Zhou, 2012). With over 150 items, the Chinese version of the CSEQ consists of four sections, as follows: students’ family background information, students’ effort and activity involvement, students’ perception of the college environment, and students’ self-report of their learning outcomes.

#### Students’ family background information

We used six items to measure students’ family background, as follows: whether their parents graduated from college (0 = no, 1 = either father or mother, 2 = both parents); whether they received parental support for tuition fees (0 = none, 1 = less than half, 2 = half, 3 = more than half, 4 = all); how many hours a week did they spend working on a job for pay inside the college (0 = more than 30 h, 1 = 21 to 30 h, 2 = 11 to 20 h, 3 = 6–10 h, 4 = less than 5 h, 5 = none: I do not have a job); how many hours a week did they spend working on a job for pay outside the college (0 = more than 30 h, 1 = 21 to 30 h, 2 = 11 to 20 h, 3 = 6–10 h, 4 = less than 5 h, 5 = none: I do not have a job); whether they possessed a computer (0 = no, 1 = yes); and whether they lived at home during the school year (0 = no, 1 = yes).

#### Students’ effort and activity involvement

Students’ effort and activity involvement are the major components of the questionnaire and include the following items/sub-components—college education opportunities and resources and frequency and extent of student activities and engagement on and off campus. We used 10 specific dimensions, including library experiences (10 items), computer and information technology (10 items), course learning (16 items), experiences with faculty (13 items), writing experiences (7 items), campus facilities (8 items), student acquaintances (12 items), clubs and organizations (5 items), topics of conversation (10 items), and scientific and quantitative experiences (10 items). All items were scored on a four-point scale (1 = never, 2 = occasionally, 3 = often, 4 = very often).

#### Students’ perception of the college environment

The college environment comprises three parts, namely scholarly and intellectual, quality of personal relations, and vocational and practical. The scholarly and intellectual part and the vocational and practical part included three items each. Students responded on a seven-point scale, with 1 indicating a weak emphasis and 7 indicating a strong emphasis. The quality of personal relations included four items that were rated on a seven-point scale, with 1 reflecting a remote, alienated, and impersonal environment, and 7 reflecting a supportive, helpful, and flexible environment.

#### Students’ self-report of their learning outcomes

In students’ self-report of their learning outcomes (25 items), we asked students to reflect on what they have gained from their college experiences and to estimate how much progress they feel they have made. These items reflect students’ holistic development, such as acquiring career information, writing clearly, understanding others, thinking logically, etc. It includes four sub-dimensions, namely, intellectual skills, personal and social development, vocational preparation, and general education. All items were scored on a four-point scale (1 = very little, 2 = some, 3 = quite a bit, 4 = very much).

### Data analysis strategies

The key to testing a proposed model is to evaluate the fit between the theoretical model and empirical data. The model plays an important role in theory development and can be operationalized through the form of semi-mathematical languages, such as structural equation modeling (SEM). The SEM is an effective way to analyze inter-relationships among different variables. It integrates factor analysis and path analysis to test the inter-relationships among different measured variables, latent variables, and error variables, thereby obtaining the direct, indirect, and total effect values of the independent variables on outcome variables (Kline, 2011). In this study, SEM, more specifically, path analysis was performed to examine the mechanisms underlying the links between various influencing factors and student learning outcomes. By using path analysis, we can compare the effects of different factors on student learning outcomes, and examine the indirect effects through the mediators (Wu, 2009). Based on the direct, indirect, and total effects, we can calculate the percentage of variation of student learning outcomes that could be explained by influencing factors.

SEM is a multivariate statistical technique to analyze the complex relationships of observed and latent variables. It can test and evaluate pre-assumed causal relationships among variables, while simultaneously accounting for measurement error. Generally, five logical steps are involved in SEM: model specification, model identification, parameter estimation, model evaluation, and model modification (Kline, 2011; Byrne, 2013). Model specification is to specify the model, and define the hypothesized relationships among multiple variables based on the theoretical model. Model identification is to check if the model is under-identified, just-identified, or over-identified. Parameter estimation is to minimize the difference between the observed and estimated population covariance matrices. Model evaluation is to assess the overall fit of the model, and the significance of particular parameters of the model. Model modification is to adjust the model for improving model fit, for example, the *post hoc* model modification. In this study, the SEM was conducted using IBM SPSS Amos version 20.0 to test the hypothesized relationship among family background, campus facilities, college environment, academic activities involvement, social activities involvement, and student learning outcomes. When developing the initial model, we adopted the hybrid model path analysis method.

The structure model refers to the linked relationship between latent variables or between latent variables and observed variables. The effect of the variables can be either direct or indirect (Wu, 2009). Family background, campus facilities, perception of the college environment, and self-reported learning outcomes were measured as observed variables. The defining feature of the measurement model is that it needs to be reflected and interpreted by a set of observed variables. Concerning the college activities scale, variables such as library experiences, computer, and information technology, course learning, writing experiences, and scientific and quantitative experiences have the highest loadings with academic activities involvement; variables such as experiences with faculty, clubs and organizations, student acquaintances, and topics of conversation have the highest loadings with social activities involvement.

The path coefficient between the latent variable and one of the observed variables was set to 1. The model’s goodness of fit was assessed by applying the Chi-square (*χ*^2^) value, comparative fit index (CFI), the Tucker–Lewis index (TLI), and the root mean square error of approximation (RMSEA). A non-significant result of *χ*^2^ test indicates a good fit for the model. However, the value of *χ*^2^ is sensitive to large sample sizes (Marsh and Balla, 1994). Hu and Bentler (1999) recommend that CFI and TLI values above 0.90, and an RMSEA value below 0.08 may be considered to be a reasonable fit for the model.

## Results

### Evaluation of the model

According to the mean, standard deviation, skewness, and kurtosis values of each variable, there is no extreme value in the distribution of the data, which is consistent with normal distribution. In the reliability and validity analyses, the internal consistency score for reliability is generally applied to test the multi-item scale. The Cronbach’s alpha coefficient is used to assess index reliability. As shown in Table 1, the alpha value of the family background is above 0.60, library experiences are above 0.70, and all other variables are above 0.80. Since the CSEQ has been revised and used in the United States and China for many years, the confirmatory factor analysis (CFA) was carried out to test the internal structure validity of the section on students’ effort and activity involvement. The results show that the ten-factor model meets the recommended criteria, *χ*^2^/df = 4.515, CFI = 0.967, TLI = 0.961, RMSEA = 0.042, indicating adequate fit of the model to the data.

**Table 1:** Descriptive statistics for study variables.

Variables		Number of items	Mean	Standard deviation	Cronbach’s alpha
Exogenous variables	Family background	6	13.64	2.929	0.675
	Campus facilities	7	17.11	3.921	0.739
	College environment	10	48.56	11.149	0.897
Endogenous variables	Academic activities				
	Library experiences	8	18.58	4.62	0.805
	Computer & information technology	9	22.24	6.122	0.863
	Course learning	12	30.84	6.014	0.824
	Writing experiences	7	16.56	4.225	0.816
	Scientific & quantitative experiences	8	20.4	4.796	0.837
	Social activities				
	Experiences with faculty	10	21.19	6.484	0.902
	Clubs& organizations	5	10.72	3.782	0.841
	Student acquaintances	12	28.6	6.897	0.871
	Topics of conversation	9	21.87	5.222	0.852
Outcome variable	Student learning outcomes	25	63.89	12.991	0.929

**Figure 2:**
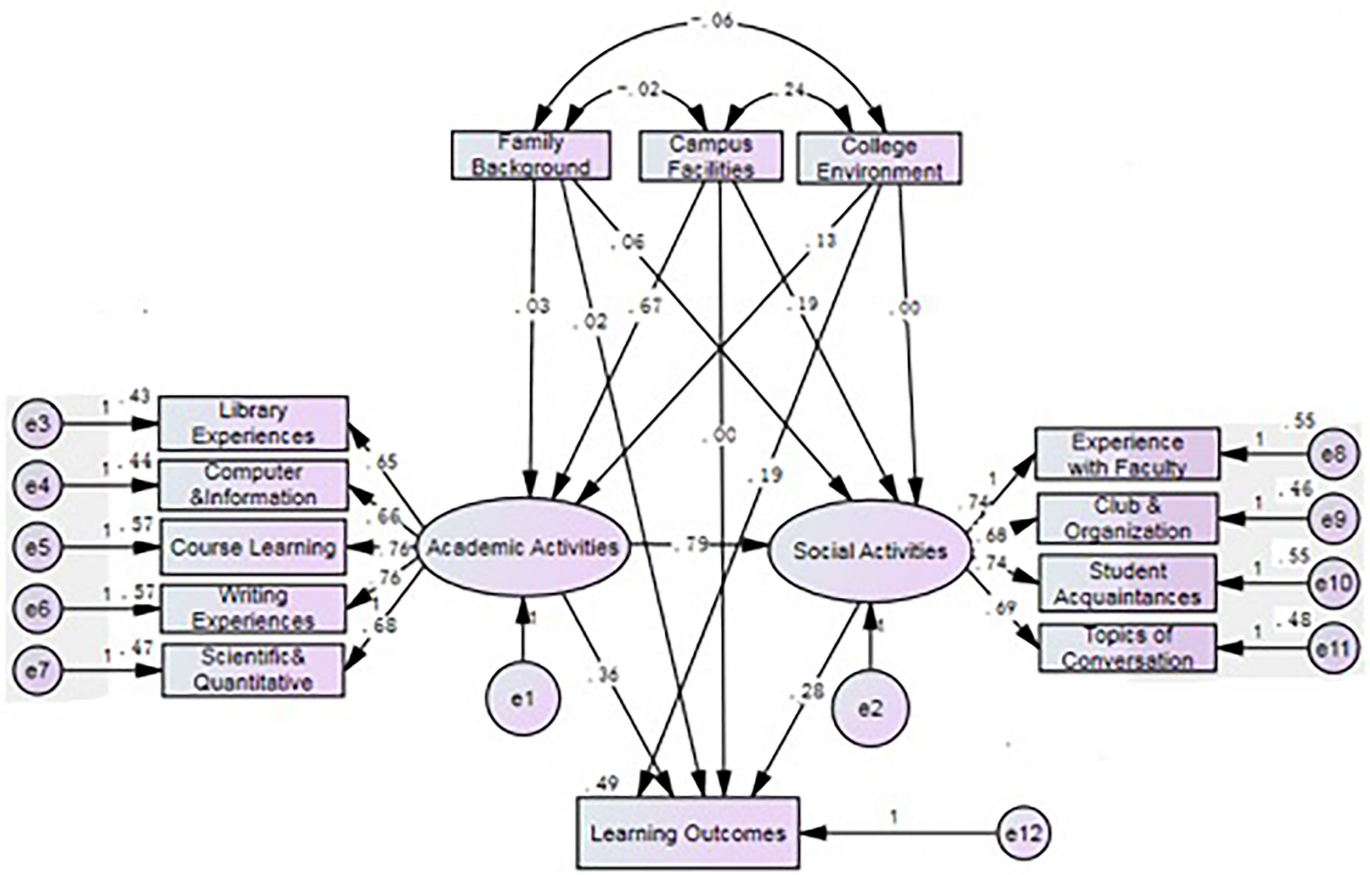
Standardized estimates from the structural equation modeling.

After conducting the CFA, the SEM was used to test the hypotheses as specified in our hypothetical model. The Chi-square for the model was significant, *χ*^2^ = 6677.98, *p* < 0.001. The goodness-of-fit indices, such as RMSEA = 0.073, CFI = 0.931, and TLI =0.901, met the criteria for adequate goodness of fit. Overall, these indices suggest a good fitness of the model. Standardized regression coefficients are presented in Figure 2. The proposed SEM model shows the impacts of the factors on the students’ self-reported learning outcomes^2^ and indicates that independent variables account for 49% of the variation of the dependent variable. The factor loadings for library experiences, computer and information technology, course learning, writing experiences, and scientific and quantitative experiences are 0.65, 0.66, 0.76, 0.76, and 0.68, respectively; the factor loadings for experiences with faculty, clubs and organizations, student acquaintances, and topics of conversation are 0.74, 0.68, 0.74, and 0.69, respectively. All factor loadings were above 0.6, indicating that two sets of observation variables reflect the measurement of the same object separately and thus reach a significant level of 0.001.

### The effects on student learning outcomes

Table 2 presents the total direct and indirect effects on student learning outcomes. The family background had the least impact on student learning outcomes. The specific value was 0.018 (*p* = 0.007). It is significantly lower than other variables and does not reach a significant level. The total effects of campus facilities (*β* = 0.432, *p* = 0.002) and academic activities (*β* = 0.574, *p* = 0.002) on student learning outcomes were higher, followed by social activities (*β* = 0.277, *p* = 0.002) and the college environment (*β* = 0.268, *p* = 0.002). The total effects of the exogenous variables (i.e., college environment, family background, and campus facilities) were relatively larger, accounting for nearly 46% of variances associated with student learning outcomes, while the total effects of endogenous variables (e.g., social activities and academic activities involvement) accounted for 54% of variances.

**Table 2:** The direct, indirect, and total effects on student learning outcomes.

		Total effect	Direct effect	Indirect effect
Effect on academic activities	Family background	0.032^**^	0.032^**^	-
	Campus facilities	0.669^**^	0.669^**^	-
	College environment	0.134^**^	0.134^**^	-
Effect on social activities	Family background	−0.031^**^	−0.056^**^	0.025^**^
	Campus facilities	0.714^**^	0.186^**^	0.528^**^
	College environment	0.099^**^	−0.007	0.106^**^
	Academic activities	0.790^**^	0.790^**^	-
Effect on learning outcomes	Family background	0.018^**^	0.015^**^	0.003
	Campus facilities	0.432^**^	−0.004	0.436^**^
	College environment	0.268^**^	0.193^**^	0.075^**^
	Academic activities	0.574^**^	0.356^**^	0.219^**^
	Social activities	0.277^**^	0.277^**^	-

* *p* < 0.05;

** *p* < 0.01;

*** *p* < 0.001.

The college environment (*β* = 0.193, *p* = 0.002), academic activities (*β* = 0.356, *p* = 0.002), and social activities (*β* = 0.277, *p* = 0.002) had relatively larger direct effects on student learning outcomes. On the other hand, family background (*β* = 0.015, *p* = 0.010) and campus facilities (*β* = −0.004, *p* = 0.760) had a small direct effect on student learning outcomes. The indirect effect of campus facilities (*β* = 0.436, *p* = 0.002) on student learning outcomes was the largest, accounting for a large proportion of the total effect. The indirect effect of academic activities (*β* = 0.219, *p* = 0.002) on student learning outcomes was also large, accounting for a significant proportion of the total effect. Campus facilities, which indirectly impact student learning outcomes, had large direct effects on academic activities (*β* = 0.669, *p* = 0.002) and social activities (*β* = 0.186, *p* = 0.002). There is a need to conduct in-depth analyses of these large indirect effects on student learning outcomes.

Involvement in academic and social activities had a large mediating effect on the relationship between campus facilities and learning outcomes. Three specific impact paths were identified and the proportion of their impact was calculated by using the mediating effect/total effect. The first path is campus facilities → academic activities → student learning outcomes; specifically (0.669*0.356) / 0.432 = 0.551, accounting for about 55% of the total effect. The second path is campus facilities → social activities → student learning outcomes; specifically (0.186*0.277)/0.432 = 0.119, accounting for about 12% of the total effect. The third path is campus facilities → academic activities→ social activities → student learning outcomes; specifically (0.669*0.790*0.277)/0.432 = 0.339, accounting for 34% of the total effect. The first path had the largest effect, while the second path has the lowest effect. In addition, social activities had a greater mediating effect on academic activities and student learning outcomes. The impact path is academic activity→ social activity→ student learning outcomes; specifically (0.790*0.277)/0.574 = 0.381, indicating that it accounted for 38% of the total effect and yielded a large mediating effect.

### The effects on the sub-dimensions of student learning outcomes

Student learning outcomes comprise four sub-dimensions—personal and social development, general education, vocational preparation, and intellectual skills. We ran SEM models for the four sub-groups of student learning outcomes separately. These models fit the data adequately. The goodness-of-fit indicators CFI and TLI values were all larger than 0.9 and RMSEA values were smaller than 0.8, which suggests a good model fit. All the influencing factors explain a significant portion variation of learning outcomes by each sub-dimension, reaching 30, 36, 29, and 40%, respectively. As shown in Table 3, both campus facilities (*β* = 0.347, *p* = 0.002) and academic activities (*β* = 0.396, *p* = 0.002) had a large total effect on personal and social development. Academic activities (*β* = 0.436, *p* = 0.002) had a large direct effect, and campus facilities (*β* = 0.256, *p* = 0.002) had a large indirect effect, through academic and social activities involvement. Social activities (*β* = −0.051, *p* = 163) had a negative direct effect on personal and social development. Concerning general education, academic activities (*β* = 0.516, *p* = 0.002) and social activities (*β* = 0.478, *p* = 0.002) had a larger total effect. Social activities (*β* = 0.478, *p* = 0.002) had a large direct effect, while campus facilities (*β* = 0.434, *p* = 0.002) had a larger indirect effect on general education through social activities and academic activities involvement. Campus facilities (*β* = −0.074, *p* = 0.002) had a negative direct effect on general education. Concerning vocational preparation, campus facilities (*β* = 0.305, *p* = 0.002) and academic activities (*β* = 0.457, *p* = 0.002) had a larger total effect. Academic activities (*β* = 0.448, *p* = 0.002) had a large direct effect but campus facilities (*β* = 0.308, *p* = 0.002) has a larger indirect effect through academic and social activities involvement. Social activities (*β* = 0.012, *p* = 0.723) had little total effect. Concerning intellectual skills, campus facilities (*β* = 0.390, *p* = 0.002) and academic activities (*β* = 0.565, *p* = 0.002) had a larger total effect. Academic activities (*β* = 0.569, *p* = 0.002) had a large direct effect, while campus facilities (*β* = 0.377, *p* = 0.002) had a larger indirect effect through academic and social activities involvement. Social activities (*β* = −0.006, *p* = 0.204) had a negative direct effect on intellectual skills.

**Table 3:** Effects on the four dimensions of student learning outcomes.

		Total effect	Direct effect	Indirect effect
Effect on personal and social development	Family background	0.026^**^	0.010	0.015^**^
	Campus facilities	0.347^**^	0.092^**^	0.256^**^
	College environment	0.246^**^	0.193^**^	0.053^**^
	Academic activities	0.396^**^	0.436^**^	−0.040
	Social activities	−0.051	−0.051	-
Effect on general education	Family background	−0.002	0.008	−0.010^*^
	Campus facilities	0.361^**^	−0.074^**^	0.434^**^
	College environment	0.193^**^	0.127^**^	0.066^**^
	Academic activities	0.516^**^	0.139^**^	0.377^**^
	Social activities	0.478^**^	0.478^**^	-
Effect on vocational preparation	Family background	0.040^**^	0.026^**^	0.014^**^
	Campus facilities	0.305^**^	−0.003	0.308^**^
	College environment	0.238^**^	0.177^**^	0.061^**^
	Academic activities	0.457^**^	0.448^**^	0.009
	Social activities	0.012	0.012	--
Effect on intellectual skills	Family background	0.046^**^	0.028^**^	0.018^**^
	Campus facilities	0.390^**^	0.013	0.377^**^
	College environment	0.230^**^	0.154^**^	0.076^**^
	Academic activities	0.565^**^	0.569^**^	−0.004
	Social activities	−0.006	−0.006	-

* *p* < 0.05;

** *p* < 0.01;

*** *p* < 0.001.

## Discussion

This study reveals the quality of undergraduate education from the perspective of student development. It focuses on the process and factors that may help to improve educational quality and student development results within the context of recent higher education reforms in China. Talent training plays a central role in the quality of higher education. The question of “What kind of person to cultivate” has always been a thread that runs through all aspects of education, and students are the key dimension of evaluation. How to assess students’ development, including the process and outcome, has become an important standard for measuring quality. In the establishment of quality system, the development of students is at the center of this system, which is among the top priorities for improving the quality of talent training. Focusing on the core status of a student, it is necessary to pay more attention to the external environment and conditions, process of students’ development, and their learning outcomes. In the evaluation of talent training quality, emphasis should be put on structure, process, and outcome quality. Therefore, quality standards should incorporate indicators reflective of structure, process, and outcome quality. HEIs should be aware of how to better serve student and more effectively promote student learning outcomes.

Although the Chinese version of the CSEQ was developed from the original US CSEQ, the BNU team made every effort to adapt the questionnaire to the Chinese educational and cultural context. We believe that the CSEQ can help break open the “black box” of the education process for Chinese undergraduates. To evaluate the quality of undergraduate education, it is important to assess students’ involvement and their learning outcomes as the assessment results could be the key indicators of learning quality and personal development (Kuh, 2009). Research on student learning outcomes can reveal students’ development following undergraduate education and the quality of the education they received in college. Specifically, the impact model of student learning outcomes shows that it can reflect the specific process, including the institutional and individual factors associated with their development, of the quality of education that undergraduate students received.

In this study, we tested a college impact model on students’ self-reported learning outcomes. Empirical data were collected from a large sample of Chinese undergraduates using the Chinese version of a survey questionnaire, the CSEQ. The research findings suggest that the proposed model fits the data adequately and the hypothesized model was verified. Proposed factors accounted for a 49% variation of self-reported learning outcomes among Chinese undergraduates. The research findings validated the premise of the questionnaire. According to Hu and Kuh (2003) and Pace (1985), when students enter college, the more resources and learning opportunities they utilize and the more they devote themselves to learning, the more benefits they are likely to obtain in terms of student learning and development. Overall, family background played a limited role in student learning outcomes. Instead, students tended to connect more with the college environment and campus facilities. Students use college as an advantageous platform to make effective use of various facilities and resources and become actively involved in academic and social activities. These behaviors promote intra-personal development and interpersonal communication skills.

In this study, the indirect effect of family background on student learning outcomes did not reach a significant level accounting for only a small portion of the total effect. Specifically, family background had a positive total and direct impact on student learning outcomes and an indirect impact on student learning outcomes through social activities involvement. However, the combined effect was smaller than the effects of other factors. Although existing research suggests that student learning outcomes differ by student group, student characteristics such as age, gender, and family income had only a slight impact on students’ development and learning outcomes (Pace, 1985; Davis and Murrell, 1993; Kuh, 2009). Except for family background, all other study variables had significant associations with the learning outcomes variable. Thus, the findings of this study reinforce past assertions that students’ involvement in social and academic activities exerts a large impact on student learning outcomes relative to their family background (e.g., parental education level and tuition support).

Students’ involvement with and participation in learning is not only a superficial behavioral display but also an identification or establishment of learning subjectivity. Christenson et al. (2013) suggest that students’ involvement with and participation in learning forms two “subjective identities”. One subjective identity is subjective valuing, which highlights the role, value, and meaning of students in their learning. The other subjective identity is subjective belonging, which highlights the bonding and connectedness between students and their classrooms/schools. Students’ involvement with and participation in learning plays a crucial role in the acquisition of learning outcomes. Empirical data on college experiences can be analyzed to identify and quantify students’ learning involvement and participation. The college impact model further verifies the associations among these variables. In our proposed model, students’ utilization of campus facilities and academic activities involvement have a significantly higher total effect on student learning outcomes than the effect of other factors. There is a significant positive correlation between participation in various specific activities and projects and student learning outcomes. Chinese students have a high level of involvement in activities such as course learning, computer and information technology, scientific and quantitative experiences, and topics of conversation.

Among the factors associated with student learning outcomes, campus facilities also exert a larger indirect effect. The campus facilities factor transmits its influence on learning outcomes through academic and social activities involvement. Thus, the mediating effects are large. The HEIs offer good external conditions and resources. However, the key to student learning and personal development is not the quantity of the resources provided but rather the extent to which the resources are utilized by students. Similar findings have been reported in other studies (Pace, 1985; Kuh and Hu, 2001; Pascarella and Terenzini, 2005). Students who participate in a wide range of activities outside the classroom perform significantly better in terms of student learning and individual development than students who participate only in academic activities (Kuh, 1995; Butler and Dawkins, 2008). Additionally, college external environments provide moral support for student development and different types of environments yield a differential impact on student learning outcomes. If students perceive that the college places emphasis on academic value, aesthetic interests, and critical thinking, students will develop in these areas. Similarly, if students experience good relationships with their peers, faculties, and administrators, they will the gainers. In the process of receiving undergraduate education, students are not engaged in mechanical and self-isolated learning for an external purpose but are establishing their value and connections between themselves and the learning environment. Thus, students’ learning process is one of the active constructions with high recognition of meaning and value.

Concerning the sub-dimensions of student learning outcomes, all factors in the model explained a high proportion of intellectual skills (40%) and general education dimensions (36%). Yet, the model explained a relatively low proportion of the personal and social development and vocational preparation dimensions, at 30 and 29%, respectively. Among the factors, campus facilities and academic activities had a greater impact on the intellectual skills, personal and social development, and vocational preparation dimensions, whereas academic and social activities had greater impacts on the general education dimension than the other factors. Similar to Davis and Murrell’s (1993) research, the present study found students’ academic and social efforts, and college environment accounted for a larger proportion of general education. In college, students participate in different types of educational activities, navigate different environments and atmospheres, and obtain different learning outcomes. As pointed out by Jonaseen and Land (1999), learning, thinking, and gaining knowledge involve relationships between people who participate in the same activities. They are constructed in the interaction between such activities and relationships and constitute a real and natural learning environment. All the factors have low explanatory power for students’ vocational preparation. This is closely related to the objectives of undergraduate education in China, which focus on general education (Guo and Han, 2018). The significance of general education is not only its intersection of subject knowledge but also the integration of ways of thinking. Built upon this goal, students’ involvement and effort emphasize students’ development of their general knowledge, which facilitates students’ academic development and the formation of intellectual skills (Zhuang and Liu, 2022). Yet, the emphasis on vocational skills and employment preparation is insufficient, which creates a skills training gap and an uncertain employment future.

Some scholars hold that learning is a personal matter and student input in learning will lead to positive learning outcomes (e.g., Choi and Rhee, 2009; Brown et al., 2013). Failures in student learning are often attributed to the students themselves. Currently, research on students’ learning experiences has become an important medium that affects student development (Kuh, 1995; Zhou and Zhou, 2012). Students’ involvement in learning is important but they need to work under supportive external conditions to be effective. In college, students become connected with the college environment and resources. This learning environment triggers students’ subjective perception of the environment, their teachers, lectures, and schoolwork. These factors as well as a positive interaction between teachers and students affect students such that they experience a cognitive state that Lv (2021) describes as “deep learning.”

The learning behavior of individual students can be considered a self-organizing system. Therefore, the individual learning mode or internal organization will gradually be generated from the individual’s interactive experience (Jorg et al., 2007). When students consider college a favorable platform, they make full use of the various resources and actively carry out academic studies and social activities to promote their cognitive development and the expansion of interpersonal communication skills. In our model, input factors such as family background, college environment, and campus facilities, along with the process factors of academic and social activities involvement, exert a positive impact on students’ self-reported learning outcomes. The extent to which students are involved in academic and social activities greatly impacts learning outcomes. The campus culture, including the institutional and micro-institutional environment, also transmits its influence on students’ effort and learning outcomes (Hu and Kuh, 2003; Cen, 2012).

In this study, the impact path model of student learning outcomes identified students’ development process and the critical factors that impact student development in college. Among them, students’ effort and process factors had a greater impact on student learning outcomes than the absolute value of input factors. Students learn in a relatively objective learning environment and are immersed in the overall context composed of various environmental elements such as knowledge, rules, media, and roles. However, concerning the subjective aspects of learning, students have formed their subjective feelings about this objective environment, including how well the teacher teaches. Were the objectives of the course presented? Does the teacher encourage students to learn actively? (Jonaseen and Land, 1999). Students are the main agents of the construction of cognitive structures and student development. Thus, as agents, they participate in activities purposefully, adapt to the external environment, integrate the available resources into their learning process, and construct experiences and outcomes to gain certain types of development (Mayer et al., 2019; Monson, 2019). When interacting with peer groups and faculty, students have their own social and cultural characteristics. They display different development characteristics in transforming the construction of social experiences into communicative competencies and personal development (Pike and Kuh, 2005; Xerri et al., 2018).

In essence, schooling is an important part of human social organization; learning in university or college is an important stage in acquiring social membership. Purposeful and meaningful learning itself is an essential characteristic of human activity. Thus, students’ learning problems have never been biologically driven behaviors based solely on individual physiological and psychological functions but group and socially driven behaviors based on specific environments, cultural traditions, and mental habits (Shi, 2022). Within better resource platform and environmental conditions provided by universities, undergraduates are more actively involved in academic activities. Given professional learning runs through the entire stage of undergraduate education, academic activities could cultivate students’ general literacy, and increase their professional knowledge and skills. Academic activities include course learning, library study, writing training, listening to lectures, etc. By participating in these activities, undergraduates can internalize the knowledge and ability they have acquired and form their own cognitive structure. According to Constructivism Learning Theory, students are active participants in their learning journey, they construct knowledge rather than just passively perceiving it. Students have their own cognitive structure, with different ways of internalizing knowledge. Thus, the favorable development of students depends not only on how much time they invest and how many books they read, but also on the rich experience inside and outside the classroom. Linking the content of classroom teaching, a student should use both school resources and social resources (Wang, 2011).

The results of this study suggest that student learning outcomes are not only the result of personal efforts but are also influenced by external campus facilities and environmental conditions. Students’ learning activities are characterized by subjectivity, a group learning mode in which students give full play to their autonomy and initiative in a specific cultural situation, and conduct multiple interactions with different types of objects. The group learning mode is jointly shaped by external environmental facilities and internal learning input.

Currently, higher education in China should focus on examining the quality of learning and cognition and view students’ learning as a behavioral input, cognitive participation, and a mixture of students’ emotions, cognition, and physical action. Concerning undergraduate student development, the following steps are required: manage uncontrollable factors, address problems and shortcomings, and promote the development of undergraduate students. For an improved undergraduate education, the college environment should encourage students’ involvement in activities; provide sufficient facilities, resources, and quality teaching; establish a supportive and interpersonal external campus environment; and foster students’ engagement with learning (Kuh, 2009; Lv, 2020; Ma and Feng, 2022).

## Implications and limitations

Given the impact of undergraduate education on students’ development, the findings of this study have some important implications for improving the quality of undergraduate education and developing students’ talents. Based on previous theoretical models and from the individual and institutional perspectives, we proposed a college impact model on the factors influencing students’ self-reported learning outcomes. The data were collected from a large sample of Chinese undergraduates using the Chinese version of the CSEQ. The research findings suggest that the proposed model fits the data adequately and the hypothesized model was verified. The proposed factors account for a 49% variation of self-reported learning outcomes among Chinese undergraduates. The average value of Chinese undergraduates’ participation in academic and social activities was significantly higher. Students’ family background played a minor role in student learning outcomes. The college environment and campus facilities not only have a direct, positive impact on student learning outcomes but also exert a larger indirect, positive impact on student learning outcomes. Both factors transmit their influence on student learning outcomes through academic and social activities involvement. Students’ involvement in social and academic activities had a large impact on learning outcomes relative to other factors. Furthermore, students’ involvement in social and academic activities mediated the association between the college environment, campus facilities, and student learning outcomes. For the sub-dimensions of student learning outcomes, all factors in our model were able to explain a high proportion of intellectual skills (40%) and general education (36%). Yet, for personal and social development and vocational preparation dimensions, the explanatory proportions were relatively low at 30 and 29%, respectively.

Firstly, we need to construct a college environment that is conducive to students’ greater involvement, participation, and engagement in learning. It is necessary to provide students with sufficient equipment and resources and establish a supportive external campus environment. Specifically, we should strengthen the academic and learning environment of HEIs, raise the requirements for receiving an academic degree, increase the external academic pressure on students, and help students to actively participate in learning. We might also need to improve the interpersonal and social communication environment of the college. The micro-environment of the college can help to prepare students for their entry into society. In the construction of the practical environment of the college, we should provide students with opportunities for social practice and internships and create conditions conducive to practical learning.

Secondly, we need to develop a joint mechanism of college and student input to enrich students’ collegiate experience. We should promote students’ holistic, behavioral, cognitive, and emotional inputs into learning and provide a range of opportunities for these inputs. We should strengthen the communication between faculty and students, establish faculty-student exchange platforms and mechanisms, and assist students to learn to cooperate and communicate and jointly construct learning tasks and goals. The educational resources of the college should be more invested in students’ activities. Informed by Astin’s (1993) concept of student involvement, colleges should encourage students to participate in academic and social situations and create favorable conditions for students to engage in meaningful learning activities.

Thirdly, there is a need to improve the undergraduate education mechanism and increase process and outcome indicators. In college, students participate in varied activities, gain learning experience, perform academically, increase their knowledge, abilities, and self-awareness, and generate learning outcomes to develop well-rounded competencies.

In the quality assessment of undergraduate education, the dimension of process and outcome should be emphasized so that students have opportunities to participate in a variety of activities. In addition, the institutional support environment should become the focus of the assessment. Meanwhile, learning outcomes should be considered as assessment indicators of education quality, which might influence an institution to transform its assessment methods. Faculty may be better able to tap students’ internal development potential, guide students’ learning behaviors, and promote students’ learning enthusiasm within the framework of a development assessment. Under jointly constructed learning goals, assessment mechanisms can be utilized to mobilize students’ cognitive and emotional investment in learning. These mechanisms can also raise students’ awareness that college learning is not merely about achieving better academic performance and receiving awards but also about cultivating a broad set of social development abilities and skills.

Our study has several limitations. First, the self-reported data from students’ self-assessment of their learning experiences and outcomes might not capture objective student learning outcomes. Standardized tests are more suited to gathering quantitative data on student development. Some scholars have pointed out that self-assessment reports may exaggerate the impact of college education on student learning outcomes and therefore should not replace standardized test scores (Pike and Kuh, 2005). In the future, it is necessary to improve research tools and add standardized tests to better reveal and explain student learning outcomes. Second, in this study, we investigated the influence of the external environment and activity factors on student learning outcomes. Although we allowed students to explore the construction of their development *via* the self-reports, we did not explore the role of a psychological mechanism in student learning outcomes; nor did we investigate student motivation, learning interests, and attitudes. Future research might further explore the effects of internal factors on student learning outcomes, including internal psychological factors such as learning motivation and attitude. Finally, due to the limitations of the research tools and analytic methods, there is a need to improve the explanatory power of the outcome variable. When studying student development, future research should pay more attention to the net impact of various factors and, with reference to causality, explain and discuss the influence of a single variable on student learning outcomes. With reference to controlling other influencing factors, the net impact of a single variable on the outcome should also be researched.

## Data availability statement

The raw data supporting the conclusions of this article will be made available by the authors, without undue reservation.

## Ethics statement

The studies involving human participants were reviewed and approved by the Minzu University of China. Written informed consent for participation was not required for this study in accordance with the national legislation and the institutional requirements.

## Author contributions

Conceptualization and design of the research were conducted by HB. HB, HY, and LL contributed to the data analyses and interpretation, RB and LL edited the manuscript. All authors contributed to the article and approved the submitted version.

## Funding

This work was supported by the Humanity and Social Science Youth Foundation of the Ministry of Education of China under Grant “A Study on the Value-Added Development and Influencing Factors of Undergraduates’ Learning Outcomes” (the grant number is 22YJC880002).

## Conflict of interest

The authors declare that the research was conducted in the absence of any commercial or financial relationships that could be construed as a potential conflict of interest.

## Publisher’s note

All claims expressed in this article are solely those of the authors and do not necessarily represent those of their affiliated organizations, or those of the publisher, the editors and the reviewers. Any product that may be evaluated in this article, or claim that may be made by its manufacturer, is not guaranteed or endorsed by the publisher.

## Footnotes

^1^The "211 Project" is a policy agenda that the Chinese government began to implement in 1993. The goal of the agenda is the construction of about 100 universities and a number of key disciplines in the 21^st^ century. Currently, the project includes 112 universities. The "985 Project" is also a policy agenda that the Chinese government began to implement in 1998. The goal of the agenda is to establish a number of the world’s advanced level, first-class university programs. Currently it includes 39 universities. In 2016, both the projects were abolished and replaced by a new program named "Double First-Class," which includes the "World-Class Universities and First-Class Disciplines Construction" strategy.

^2^From the square of the multivariate correlation coefficient next to the process variable and the result variable, the value next to the result variable is 0.49.

## References

[ref1] Astin A. W. (1993). What matters in college? Four critical years revisited. San Francisco, CA: Jossey-Bass.

[ref2] Bai H. Liu Y. (2017). Research of college campus environment impact on undergraduate students’ learning outcomes. Educ. Rev. 4, 126–130.

[ref3] Borden V. M. (2011). Accountability for student learning: views from the inside out and the outside in. Alternation. 2, 317–342.

[ref4] Brown G. A. Bull J. Pendlebury M. (2013). Assessing student learning in higher education. New York, NY: Routledge.

[ref5] Butler K. L. Dawkins P. W. (2008). The impact of a "healthy youth" learning community on student learning outcome measures. J. Negro. Educ. 3, 264–270.

[ref6] Byrne B. M. (2013). Structural Equation Modeling with AMOS: Basic Concepts, Applications, and Programming. New York, NY: Routledge.

[ref7] Casey K. M. (2004). Greater expectation: teaching and assessing for academic skills and knowledge in the general education history classroom. Hist. Teach. 37, 171–181. doi: 10.2307/1555650

[ref8] Cen Y. (2012). Growth as product and as process: student learning outcomes attained through college experiences in China. Dissertation/doctor’s thesis. Bloomington, IN: Indiana University of Bloomington.

[ref9] Chickering A. W. Gamson Z. F. (1987). Seven principles for good practice in undergraduate education. AAHE Bull. 3, 3–7.

[ref10] Clever M. Miller K. S. (2019). I understand what they’re going through: how socioeconomic background shapes the student service-learning experience. Teach. Sociol. 47, 204–218. doi: 10.1177/0092055X19832646

[ref11] Choi J. Rhee B. S. (2009). Examining factors related to college students' learning outcomes: focusing effects of college. J. Educ. Adm. 1, 151–164.

[ref12] Christenson S. L. Reschly A. L. Wylie C. (2013). Handbook of Research on Student Engagement. New York: Springer.

[ref13] Davis T. M. Murrell P. H. (1993). A structural model of perceived academica, personal and vocational gains related to college student responsibility. Stud. High. Educ. 34, 267–289. doi: 10.1007/BF00991846

[ref15] Feldman K. A. Newcomb T. A. (1969). The Impact of College on Students: An Analysis of Four Decades of Research. San Francisco: Jossey-Bass.

[ref16] Guo H. Han T. (2018). Empirical study on the impact of undergraduates’ research experience on learning gains. Educ. Res. 6, 60–69.

[ref17] Hill H. C. Chin M. (2018). Connections between teachers' knowledge of students, instruction, and achievement outcomes. Am. Educ. Res. J. 5, 1076–1112. doi: 10.3102/2F0002831218769614

[ref18] Howard J. Zoeller A. (2007). The role of the introductory sociology course on students' perceptions of achievement of general education goals. Teach. Sociol. 35, 209–222. doi: 10.1177/0092055X0703500301

[ref19] Hu B. (2006). The evolution of quality outlook of higher education. Educ. Res. 11, 24–28.

[ref20] Hu L. Bentler P. M. (1999). Cutoff criteria for fit indexes in covariance structure analysis: conventional criteria versus new alternatives. Struct. Equ. Model. 6, 1–55. doi: 10.1080/10705519909540118

[ref21] Hu S. P. Kuh G. D. (2003). Maximizing what students get out of college: testing a learning productivity model. J. Coll. Stud. Dev. 44, 185–203. doi: 10.1353/csd.2003.0016

[ref23] Jonaseen D. H. Land S. (1999). Theoretical Foundations of Learning Environments. Hillsdale, MI: Lawrence Erlbaum.

[ref24] Jorg T. Davis B. Nickmans G. (2007). Towards a new, complexity science of learning and education. Rev. Educ. Res. 2, 145–156. doi: 10.1016/j.edurev.2007.09.002

[ref25] Keen C. Hall K. (2009). Engaging with difference matters: longitudinal student outcomes of co-curricular service-learning programs. J. High. Educ. 80, 59–79. doi: 10.1080/00221546.2009.11772130

[ref26] Kline R. B. (2011). Principles and Practice of Structural Equation Modeling. In Methodology in the Social Sciences (3rd). New York: Guilford Press.

[ref27] Kuh G. D. (1995). The other curriculum: out-of-class experiences associated with student learning and personal development. J. High. Educ. 66, 123–155. doi: 10.1080/00221546.1995.11774770

[ref28] Kuh G. D. Pace R. Vesper N. (1997). The development of process indicators to estimate student gains associated with good practices in undergraduate education. Res. High. Educ. 4, 435–454. doi: 10.1023/A:1024962526492

[ref29] Kuh G. D. Hu S. P. (2001). Learning productive at research universities. J. High. Educ. 72, 1–29. doi: 10.2307/2649131

[ref30] Kuh G. D. (2009). The national survey of student engagement: conceptual and empirical foundations. New Dir. Inst. Res. 2009, 5–20. doi: 10.1002/ir.283

[ref31] Laird T. F. Cruce T. M. (2009). Individual and environmental effects of part-time enrollment status on student-faculty interaction and self-reported gains. High. Educ. 80, 290–314. doi: 10.1080/00221546.2009.11779014

[ref32] Lu G. Li L. (2020). The questionnaire design and basic characteristics of undergraduates’ course experience: a survey of 2018 graduates in Shaanxi province. J. East China Normal Univ. Educ. Sci. Edn. 11, 78–89. doi: 10.16382/j.cnki.1000-5560.2020.11.006

[ref33] Lucero J. E. Gallego S. Hedgepeth C. Sanders D. (2021). Structure and characteristics for successful outcomes: a review of community college internships programs. Community Coll. J. Res. Pract. 45, 103–116. doi: 10.1080/10668926.2019.1647901

[ref34] Lv L. (2020). Research on the Chinese undergraduates’ learning engagement and its development effect in top talents plan: based on the survey of 12 top talents plan universities in China. Res. Educ. Dev. 13, 26–38. doi: 10.14121/j.cnki.1008-3855.2020.z1.006

[ref35] Lv L. (2021). Top students’ deep learning motivation: a study focusing on two kinds of interests: based on the survey of 12 “top talents plan” universities in China. J. Nanjing Normal Univ. 2, 76–88.

[ref36] Ma L. Feng Q. (2022). The influence of high-impact educational practices on undergraduates critical thinking ability: an empirical study based on a double first-class construction university. China High. Educ. Res. 5, 72–79. doi: 10.16298/j.cnki.1004-3667.2022.05.12

[ref37] Marsh H. W. Balla J. (1994). Goodness of fit in confirmatory factor analysis: the effects of sample size and model parsimony. Qual. Quant. 28, 185–217. doi: 10.1007/BF01102761

[ref38] Mahan D. M. (2010). The four-year experience of first generation students at a small independent university: Engagement, Student Learning and satisfaction. Dissertation/doctor’s thesis. Louisville, KY: University of Louisville.

[ref39] Mayer B. Blume A. Black C. Stevens S. (2019). Improving student learning outcomes through community-based research: the poverty workshop. Teach. Sociol. 47, 135–147. doi: 10.1177/2F0092055X18818251

[ref40] Mckinney K. Medvedeva M. Vacca K. Malak J. (2004). Beyond the classroom: an exploratory study of out-of-class learning in sociology. Teach. Sociol. 32, 43–60. doi: 10.1177/2F0092055X0403200106

[ref41] Ministry of Education of the People’s Republic of China. (2021). 2020年全国教育事业发展统计剬报. http://www.moe.gov.cn/jyb_sjzl/s5990/202111/t20211115_579974.html (Accessed November 15, 2021).

[ref42] Monson R. A. (2019). Do they have to like it to learn from it? Students’ experiences, group dynamics, and learning outcomes in group research projects. Teach. Sociol. 47, 116–134. doi: 10.1177/0092055X18812549

[ref43] Osegura L. Rhee B. S. (2009). The influence of institutional retention climates on student persistence to degree completion: a multilevel approach. Res. High. Educ. 50, 546–569. doi: 10.1007/s11162-009-9134-y

[ref44] Pace C. R. (1979). Measuring Outcomes of College: Fifty Years of Findings and Recommendations for the Future. San Francisco, CA: Jossey-Bass.

[ref45] Pace C. R. (1985). Perspectives and problems in student outcomes research. New Dir. Inst. Res. 1985, 7–18. doi: 10.1002/ir.37019854703

[ref46] Pak C. S. (2020). Exploring the long-term impact of service-learning: former students of spanish revisit their community engagement experiences. Hispania 103, 67–85. doi: 10.1353/hpn.2020.0004

[ref47] Pascarella E. T. (1985). College Environmental Influences on Learning and Cognitive Development: A Critical Review and Synthesis. New York: Agathon Press.

[ref48] Pascarella E. T. Terenzini P. T. (2005). How College Affects Students: A Third Decade of Research. San Francisco, CA: Jossey-Bass.

[ref49] Pascarella E. T. Blaich C. Maitin G. L. (2011). How robust are the findings of academically adrift? High. Educ. 43, 20–24. doi: 10.1080/00091383.2011.568898

[ref50] Pike G. R. Kuh G. D. (2005). A typology research of student engagement for American college and universities. Res. High. Educ. 46, 185–209. doi: 10.1007/s11162-004-1599-0

[ref51] Tinto V. (1975). Dropout from higher education: a theoretical synthesis of recent research. Rev. Educ. Res. 45, 89–125. doi: 10.3102/00346543045001089

[ref52] Qin X. Lv L. (2022). Learning involvement and critical thinking in the cultivation of top-notch innovative personnel: an empirical study based on 12 universities with top-notch talent cultivation projection China. Jiangsu High. Educ. 1, 73–82. doi: 10.13236/j.cnki.jshe.2022.01.010

[ref53] Shi J. (2022). Chinese college students' subjective learning: studying local issues from a global perspective. China High. Educ. Res. 3, 99–104. doi: 10.16298/j.cnki.1004-3667.2022.03.15

[ref54] Shi Q. Wang A. (2010). Quality of higher education: from epistemology to axiology. J. Xiamen Univ. 2, 72–78.

[ref55] Shi Y. Ma Y. MacLeod J. Yang H. H. (2020). College students’ cognitive learning outcomes in flipped classroom instruction: a meta-analysis of the empirical literature. J. Comput. Educ. 7, 79–103. doi: 10.1007/s40692-019-00142-8

[ref56] Terenzini P. T. Pascarella E. T. Blimling G. S. (1999). Students' out-of-class experiences and their influence on learning and cognitive development: a literature review. J. Coll. Stud. Dev. 40, 610–623.

[ref57] Topping K. J. (1996). The effectiveness of peer tutoring in further and higher education: a typology and review of the literature. High. Educ. 32, 321–345. doi: 10.1007/BF00138870

[ref58] Wang S. (2011). The impact on student learning of student engagement in research universities: Based on “NSSE-China” 2009 data analysis. Tsinghua Journal of Education 4, 25–31. doi: 10.14138/j.1001-4519.2011.04.006

[ref59] Weidman J. (1989). Undergraduate Socialization: a Conceptual Approach. New York: Agathon Press.

[ref60] Wu M. (2009). Structural Equation Modeling--Manipulation and Application. Chongqing: Chongqing University Press.

[ref61] Xerri M. J. Radford K. Shacklock K. (2018). Student engagement in academic activities: a social support perspective. High. Educ. 75, 589–605. doi: 10.1007/s10734-017-0162-9

[ref62] Yu H. S. Ko J. W. Lim H. N. (2011). Examining learning experiences influencing on the communication skills and high-order thinking skills. J. Educ. Adm. 4, 319–337.

[ref63] Zhang Q. Tang H. (2021). Course learning and undergraduates added value of critical thinking ability. J. High. Educ. 8, 79–88.

[ref64] Zhuang Y. Liu Y. (2022). The influencing factors of lower grade undergraduates’ innovation capability in science and technology based on structural equation model. China High. Educ. Res. 4, 51–56. doi: 10.16298/j.cnki.1004-3667.2022.04.08

[ref65] Zhou T. Zhou Z. (2012). An exploratory study on factors influencing the development of college students. Fudan Univ. Educ. Rev. 3, 48–55. doi: 10.13397/j.cnki.fef.2012.03.014

